# Faster relaxation of nonphotochemical quenching in C4 than in C3 species

**DOI:** 10.1073/pnas.2621033123

**Published:** 2026-09-18

**Authors:** Lucía Arce Cubas, Asuka Nakamura, Lauana Pereira de Oliveira, Richard L. Vath, R. Shawn Abrahams, Julia Walter, Cristina Rodrigues Gabriel Sales, Emmanuel L. Bernardo, Yuri Nakajima Munekage, Stephen P. Long, Johannes Kromdijk

**Affiliations:** ^a^ Department of Plant Sciences, University of Cambridge, Cambridge CB2 3EA, United Kingdom; ^b^ Photosynthesis and Food Security, Carl R. Woese Institute for Genomic Biology, University of Illinois at Urbana-Champaign, Urbana, IL 61801; ^c^ Department of Bioscience, School of Science and Technology, Kwansei Gakuin University, Sanda 669-1337, Japan; ^d^ Department of Plant Biology, University of Illinois at Urbana-Champaign, Urbana, IL 61801; ^e^ Crop Physiology Division, Institute of Crop Science, College of Agriculture and Food Science, University of the Philippines Los Baños, Laguna 4031, Philippines

**Keywords:** photosynthesis, C4 pathway, nonphotochemical quenching, NPQ, photoprotection

## Abstract

Acceleration of photoprotective nonphotochemical quenching (NPQ) responses to changes in light intensity has been suggested as a strategy to enhance crop yield. Our current understanding of NPQ overwhelmingly comes from C3 species, and despite many key crops being C4, separating the impact of photosynthetic pathway from species-specific effects is complicated by the large evolutionary distance between most C3 and C4 species. By conducting a series of experiments on C3 and C4 species from *Alloteropsis, Flaveria*, and *Cleome*, genera which encompass both pathways, we controlled for phylogenetic distance and showed that NPQ relaxation is significantly faster in C4 than in C3 species. This trend is supported further by a meta-analysis compiling NPQ data on C3 and C4 species from intraspecific published studies and new measurements. Analysis of dynamic NPQ responses in C3 and C4 leaves infiltrated with inhibitors revealed that faster relaxation observed in C4 species is driven by a greater contribution of energy-dependent quenching (qE) to overall NPQ. This C4-associated enhancement of qE in turn is linked to altered regulation of lumen pH in C4 species, reflecting increases in cyclic electron flow and membrane proton conductivity to meet the increased adenosine triphosphate (ATP) demands of the C4 pathway. Altogether, our results indicate that NPQ responses in C4 species may already be optimized to maintain high photosynthetic efficiency in fluctuating light conditions. Given the intrinsically faster NPQ in C4 photosynthesis, further acceleration of NPQ may have limited scope to enhance crop photosynthetic efficiency.

## Significance

Acceleration of nonphotochemical quenching has been proposed as a means to enhance crop photosynthetic efficiency in C3 species, but whether this strategy has potential in C4 species, which include several major crops, remains unclear. Here, we show that across three phylogenetically paired C3 and C4 species, nonphotochemical quenching (NPQ) relaxation is significantly faster in species with the C4 pathway. This trend is further supported by meta-analysis across a wider range of species. As a result, accelerating the rate of NPQ relaxation in C4 crops may have a more limited scope to enhance photosynthesis.

Nonphotochemical quenching (NPQ) refers to a collection of photoprotective mechanisms wherein excess light energy in photosystem II (PSII) is dissipated as heat, preventing overexcitation and excessive formation of reactive oxygen species that would otherwise damage the photosynthetic machinery (1). NPQ components operate at different timescales and likely involve conformational changes in PSII-associated antennae that trigger the quenched state (2). Energy-dependent quenching (qE) is activated within seconds to minutes by lumen acidification (3), which also triggers xanthophyll cycle enzyme violaxanthin de-epoxidase (VDE) to convert violaxanthin to zeaxanthin, enhancing qE (4). Zeaxanthin accumulation further supports a qE-independent quenching component (qZ) that activates and recovers over minutes to hours (5). Even more sustained quenching (qH) relies on the plastid lipocalin LCNP and is negatively regulated by suppressor of quenching SOQ1 (6). Finally, photoinhibitory quenching (qI) is associated with photodamage and requires de novo synthesis of the D1 protein for recovery (7).

Our molecular understanding of NPQ as detailed above overwhelmingly comes from C3 species, where NPQ relaxation has been found to significantly lag behind changes in irradiance, temporarily lowering photosynthetic efficiency and leading to substantial losses in “foregone” canopy carbon assimilation (8). Accelerating recovery from photoprotection represents a promising strategy for improving crop yield in C3 species (9, 10), yet while many key crops are C4 (11), the specifics of the C4 NPQ response remain largely unknown (12).

In C3 photosynthesis, CO_2_ is directly fixed in mesophyll (M) chloroplasts by ribulose-1,5-biphosphate carboxylase/oxygenase (Rubisco) into 3-carbon compound 3-phosphoglycerate (3-PGA). Higher photosynthetic rates are generally found in C4 species, where a carbon concentrating mechanism (CCM) enhances photosynthesis by suppressing RuBP oxygenation and concomitant photorespiration (13). The C4 pathway operates between morphologically distinct M and bundle sheath (BS) cells, typically arranged in “Kranz” anatomy: CO_2_ is initially converted to bicarbonate in the M and fixed into 4-carbon oxaloacetate that is further reduced or transaminated into malate or aspartate before diffusion into the BS, where decarboxylating enzymes release CO_2_ around Rubisco and into the C3 cycle (14). Different C4 “subtypes” use different decarboxylases, often in combination (15), and have additional cell and subtype-specific ATP requirements for the regeneration of CCM biochemical intermediates (16–18). The ATP:NADPH ratio generated by linear electron flow (LEF) from PSII to PSI is insufficient to satisfy the demands of the C3 cycle, and the additional ATP demands by C4 metabolism further the imbalance (19). Cyclic electron flow (CEF) helps balance energy budgets by recycling electrons around PSI back to plastoquinone (PQ), contributing to the proton motive force (*pmf*) that powers ATP synthesis without concurrent production of NADPH (20). Considerably higher ratios of PSI:PSII (21, 22) and CEF:LEF (23) are found in C4 vs. C3 species, reflecting the ATP cost of the C4 pathway.

The functional differences between C3 and C4 photosynthesis are likely to affect NPQ. In C3 species, CEF plays a major photoprotective role by contributing to ∆pH and thus qE activation, with NPQ being severely reduced in CEF-defective mutants (24, 25). The higher CEF:LEF ratios found in C4 species could therefore have implications for qE regulation. In C3 species, CEF is predominantly mediated by proton gradient regulation 5 (PGR5) and PGR5-like photosynthetic phenotype 1 (PGRL1), with a minor contribution by chloroplast NADH dehydrogenase-like complex (NDH) in low and fluctuating light (26–28). In C4 species, although PGR5/PGRL1 remains important, recent studies show CEF occurs primarily via NDH (29–31). Notably, NPQ amplitude is lower in C4 PGR5 and PGRL1-deficient mutants, but higher in NDH mutants (30, 31). Based on these results, it was suggested that PGR5/PGRL1 could play a similar photoprotective role in C4 photosynthesis as in C3 species, with NDH mainly contributing to supplementing ATP production (31), but the effect of CEF pathway interplay on C4 NPQ remains unclear. Finally, NPQ modulation is also regulated by the proton conductivity of the thylakoid membrane (gH^+^, indicative of ATP synthase activity), as changes to gH^+^ affect *pmf* formation and dissipation (32). Higher ATP demand in C4 species results in faster turnover of inorganic phosphate and substrate availability for ATP synthase (33), potentially resulting in altered control of ∆pH-dependent NPQ relative to C3 photosynthesis.

The present work aimed to characterize differences in NPQ relaxation between C3 and C4 species using a combination of spectroscopic, chemical, and molecular approaches, including C4 CEF mutants. To account for the strong confounding effect of phylogenetic distance (34), we compared phylogenetically linked pairs of C3 and C4 species from three evolutionarily distinct genera, and conducted a meta-analysis combining published data with new measurements of species operating C3 and C4 pathways.

## Results

### Differences in NPQ Relaxation between C3 and C4 Species.

**Fig. 1.**
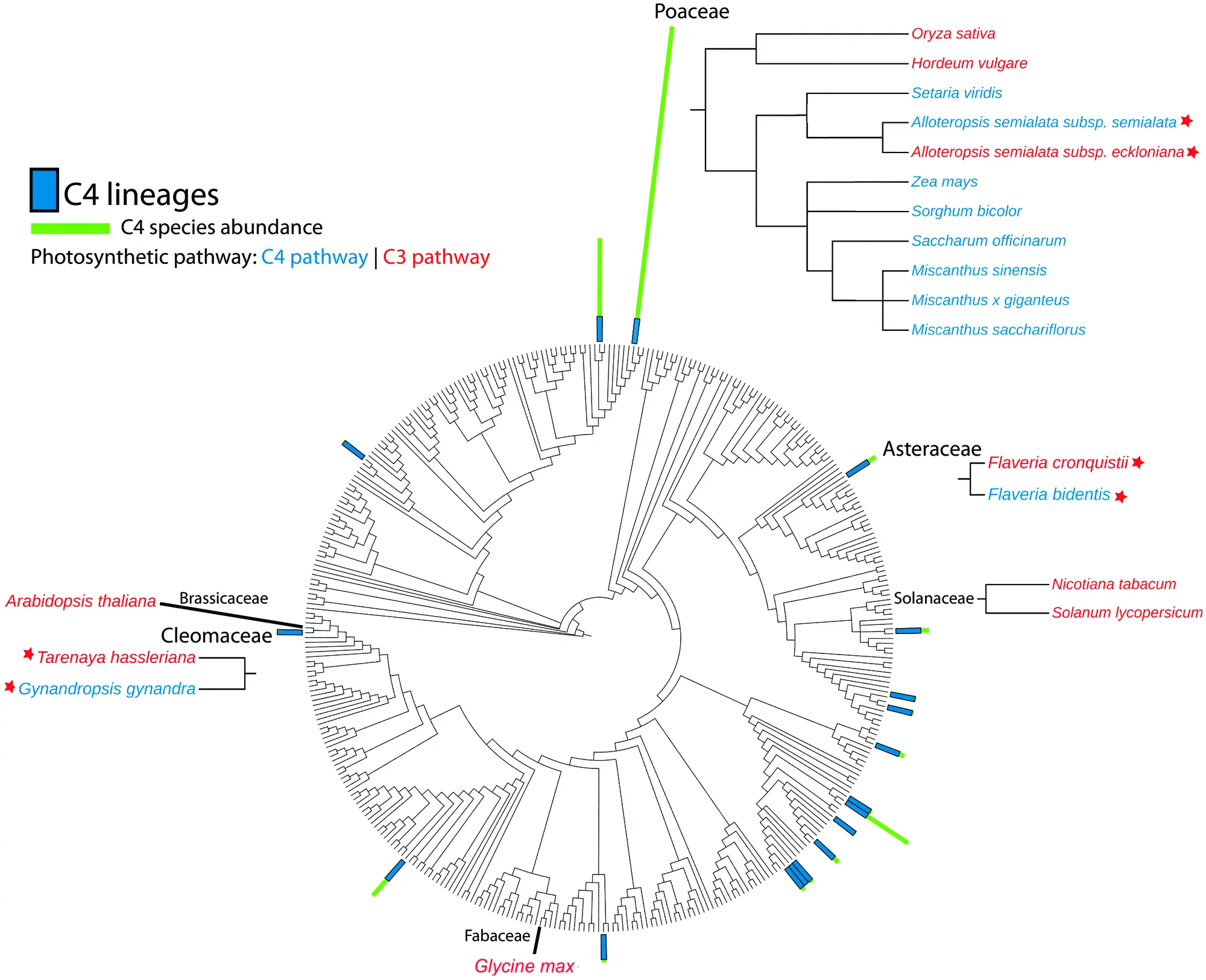
Phylogenetic tree of angiosperm families (based on 72 to 74) showing the distribution of C4 origins (blue squares). The number of C4 species in each family (45) is represented by the length of green bar. The species used in three phylogenetically controlled comparisons in this study are indicated (red star), as well as the extended list of C3 and C4 species included in the meta-analysis (Fig. 2*C*) with the exception of the bryophyte Marchantia.

Comparisons between C3 and C4 photosynthesis are challenging due to the strong confounding effects of evolutionary distance (34–37) and the bias toward PACMAD NADP-ME C4 lineages (38). We controlled for phylogenetic variation at the genus level, selecting C3 and C4 species from three of the four (35, 39) known genera containing both pathways: *Alloteropsis* (*C3 A. semialata KWT, C4 A. semialata MDG*), *Flaveria* (*C3 F. cronquistii, C4 F. bidentis*), and *Cleome* (*C3 Tarenaya hassleriana, C4 Gynandropsis gynandra*). This experimental design allowed for a comprehensive comparison between photosynthetic types by reducing phylogenetic variation between C3 and C4 pairs while maintaining evolutionary diversity: The selected species represent both monocots (*Alloteropsis*) and dicots (*Flaveria*, *Cleome*), three independent C4 origins (40, 41), and all three major C4 decarboxylating enzymes: NADP-ME/PEPCK in C4 *A. semialata MDG* (42), NADP-ME in C4 *F. bidentis* (43), and NAD-ME in C4 *G. gynandra* (44). Fig. 1 situates these species within the angiosperm family tree and illustrates the evolutionary distance that exists even at the clade level between other commonly studied C3 and C4 taxa.

**Fig. 2.**
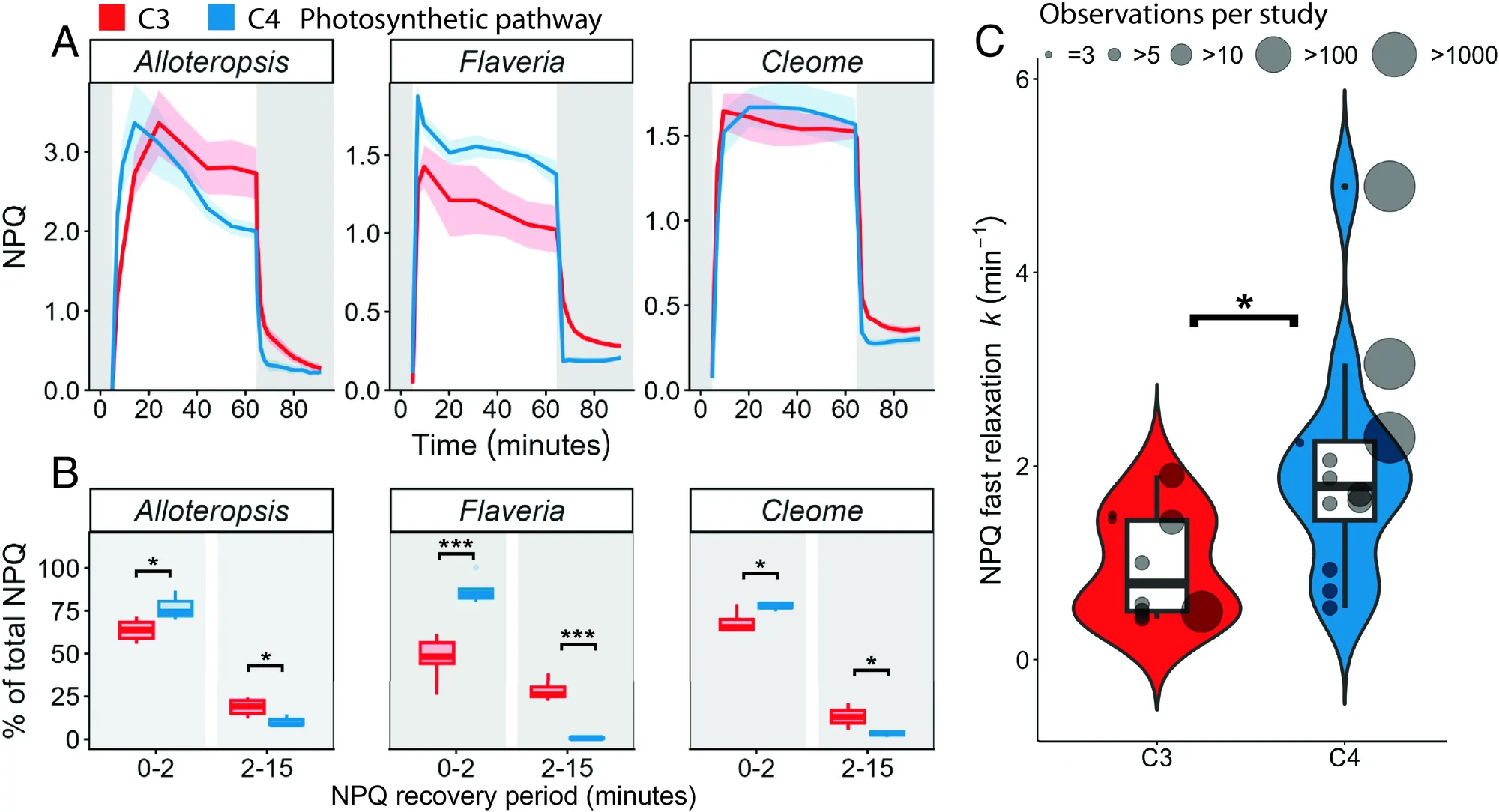
Differences in NPQ relaxation between phylogenetically linked C3 and C4 species. (*A*) NPQ during 1 h of illumination at 600 µmol m^−2^ s^−2^ PFD followed by 30 min of darkness in three pairs of phylogenetically linked C3 and C4 species. Ribbons represent SEM (n = 5). (*B*) NPQ recovery during the first 2 min (0 to 2) as a percentage of NPQ at the end of the light period. (*C*) Violin plot of first-order NPQ relaxation constants of diverse C3 and C4 species, combining new data with existing published studies on NPQ relaxation. Each study is represented by a dark circle, with symbol size being proportional to the number of recorded observations within each dataset. *SI Appendix*, Table S3 includes the specific values, species, data sources, and experimental conditions across these datasets. Asterisks indicate significant differences between C3 and C4 species found by one-way ANOVA (*P ≤ 0.05, **P ≤ 0.01, ***P ≤ 0.001).

NPQ induction and relaxation responses were measured during a 1-h photoperiod at saturating light intensity (600 µmol m^−2^ s^−1^ PFD), followed by 25 min of darkness (Fig. 2*A*). While no clear trends were present during induction, there was a clear differentiation of relaxation by photosynthetic pathway. Given current strategies to accelerate NPQ for increased photosynthetic efficiency rely on accelerated recovery (9, 10, 46, 47), we focused on NPQ following the light-to-dark transition. NPQ relaxation was faster in all C4 species relative to their C3 counterparts, resulting in significantly lower NPQ across the dark period. Integrated NPQ was 40% lower in C4 *Alloteropsis semialata MDG* than in C3 *A. semialata KWT*, 33% lower in C4 *Flaveria bidentis* than in C3 *Flaveria cronquistii*, and 22% lower in C4 *G. gynandra* than in C3 *T. hassleriana* across the dark recovery period (values and statistics in *SI Appendix*, Table S1). This experiment was also conducted under excess light (1,500 µmol m^−2^ s^−1^ PFD) where the same pattern of faster C4 NPQ relaxation was also observed (*SI Appendix*, Fig. S1).

The NPQ relaxation kinetics of C3 and C4 species were underpinned by significant differences in NPQ composition (Fig. 2*B* and *SI Appendix*, Table S2). NPQ components were analyzed by separating NPQ relaxation into different timescales of deactivation (1, 2) expressed as a function of total NPQ. Fast-relaxing components (0 to 2 min) constituted a significantly greater proportion of NPQ in all C4 species than in their C3 pairs and conversely, NPQ components with a slower recovery period (2 to 15 min) were a larger part of NPQ in C3 species, contributing to a more exponential decay.

To establish whether these results were representative of other species and light treatments, we conducted a meta-analysis of NPQ recovery kinetics across published C3 and C4 species (9, 48–54), complemented with new measurements. The final analysis included twenty independent datasets of fourteen additional C3 and C4 species (*SI Appendix*, Table S3, species also included in Fig. 1). Supporting our findings from the paired species comparisons, the meta-analysis found NPQ relaxation constants were significantly faster in C4 (*k* = 1.96 ± 0.33 min^−1^) than in C3 species (*k* = 0.97 ± 0.17 min^−1^), demonstrating that this difference is commonly found across a wide range of species, growth, and experimental conditions (Fig. 2*C*).

### Effects of Chemical Inhibition of ΔpH and Xanthophyll-Dependent Components.

**Fig. 3.**
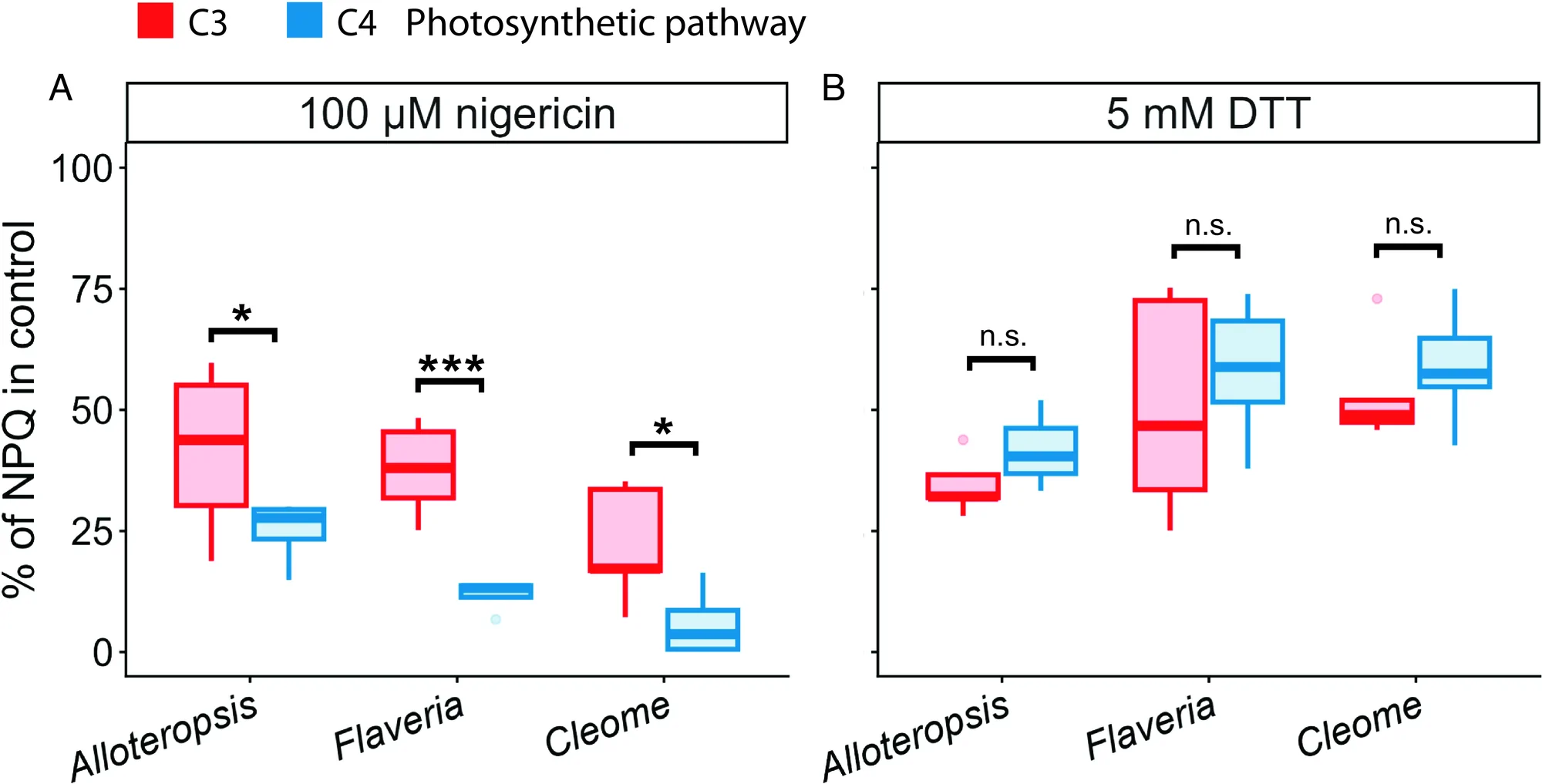
Effect of ΔpH disruption and xanthophyll cycle inhibition on NPQ in phylogenetically linked C3 and C4 species. Plots show AUC of NPQ induction in leaves infiltrated with (*A*) 100 µM nigericin to collapse the proton gradient and (*B*) in leaves infiltrated with 5 mM dithiothreitol to inhibit zeaxanthin formation via VDE activity. AUCs are normalized to mock-infiltrated control leaves. Asterisks indicate significant differences between C3 and C4 species based on one-way ANOVA (n = 5, *P ≤ 0.05, **P ≤ 0.01, ***P ≤ 0.001).

Fast-relaxing NPQ components include ΔpH-sensitive and xanthophyll-dependent qE in the first 2 min, and qZ dissipation in 2 to 15 min (2). To identify the elements responsible for NPQ differences between C3 and C4 species, leaves were infiltrated with H^+^/K^+^ antiporter nigericin to collapse the proton gradient, or with dithiothreitol (DTT) to inhibit VDE activity and thereby zeaxanthin formation via the xanthophyll cycle. To limit off-target effects, various concentrations were tested prior to the experiment—the lowest concentration at which an effect on NPQ could be observed without affecting *F_v_/F_m_* was then chosen (*SI Appendix*, Fig. S3). The integrated NPQ of treated leaves during the light period was compared to mock-infiltrated controls to assess NPQ dependence on both mechanisms (Fig. 3, full traces in *SI Appendix*, Fig. S2). Nigericin infiltration resulted in significantly greater suppression of NPQ in C4 than in C3 species (Fig. 3*A* and *SI Appendix*, Table S4), indicating higher reliance on ΔpH for C4 NPQ responses: 75 ± 3% reduction in C4 *A. semialata MDG* vs. 58 ± 9% in C3 *A. semialata KWT*, 88 ± 2% in C4 *F. bidentis* vs. 62 ± 4% in C3 *F. cronquistii*, and 94 ± 3% in C4 *G. gynandra* vs. 68 ± 3% in C3 *T. hassleriana*. Although both qE and xanthophyll de-epoxidation depend on ΔpH, the lack of significant differences in NPQ suppression by 5 mM DTT in any C3 and C4 pairs (Fig. 3*B*) suggests that differences in C4 NPQ mostly stem from ΔpH-sensitive qE rather than qZ or xanthophyll-dependent qE.

### Effects of the Removal of Photosynthetic and Photorespiratory Electron Sinks.

**Fig. 4.**
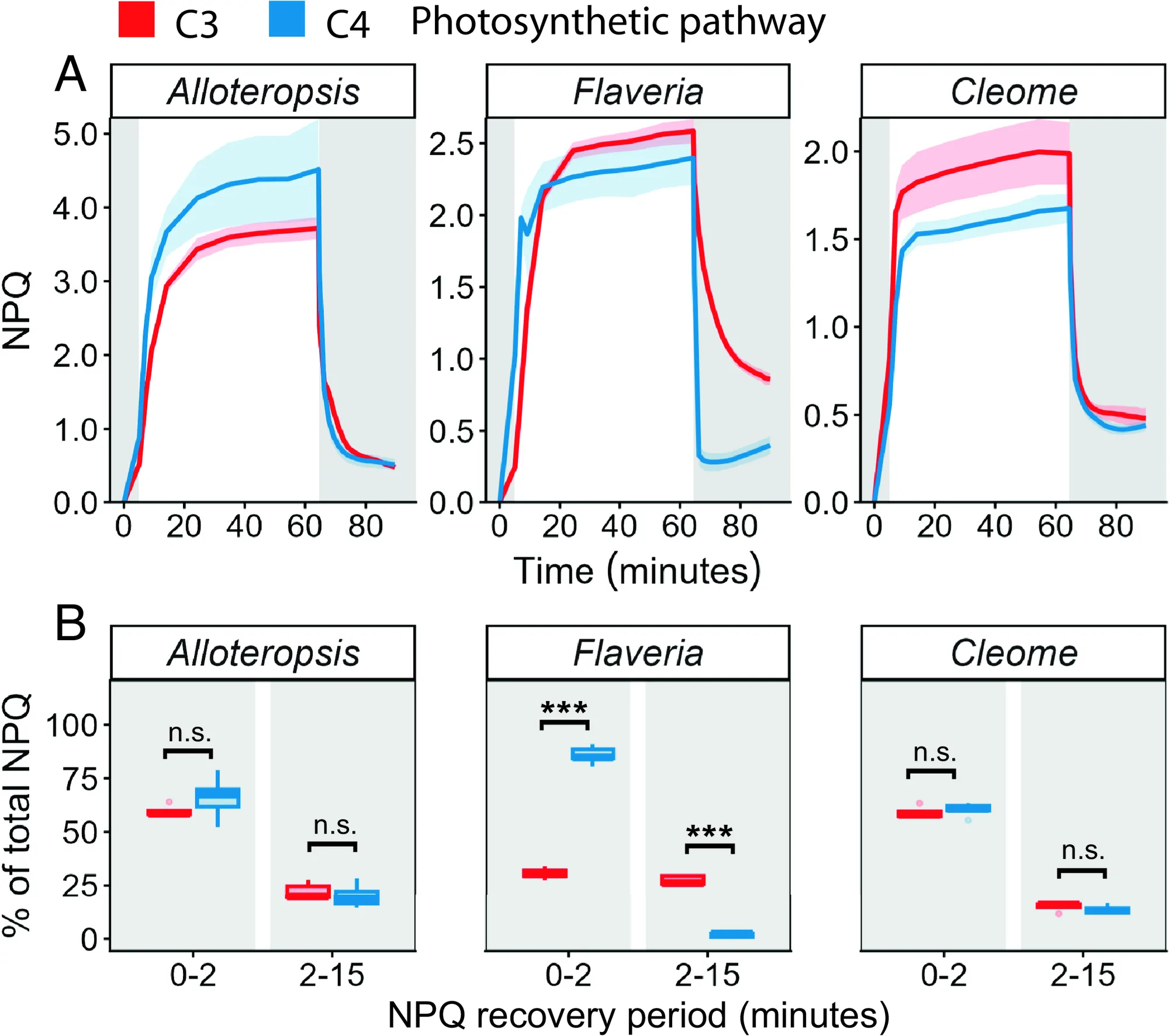
Differences in NPQ in phylogenetically linked C3 and C4 species when suppressing photosynthesis and photorespiration with 2% O_2_ and 0 ppm CO_2_ air. (*A*) NPQ levels during 1 h of illumination at 600 µmol m^−2^ s^−2^ PFD followed by 25 min of darkness. Ribbons represent SEM. (*B*) NPQ recovery amplitude relative to NPQ at the end of the illumination period, separated between recovery during 0 to 2 min of darkness or the subsequent 2 to 15 min of darkness. Asterisks indicate significant differences between C3 and C4 species based on one-way ANOVA (n = 5, *P ≤ 0.05, **P ≤ 0.01, ***P ≤ 0.001).

**Fig. 5.**
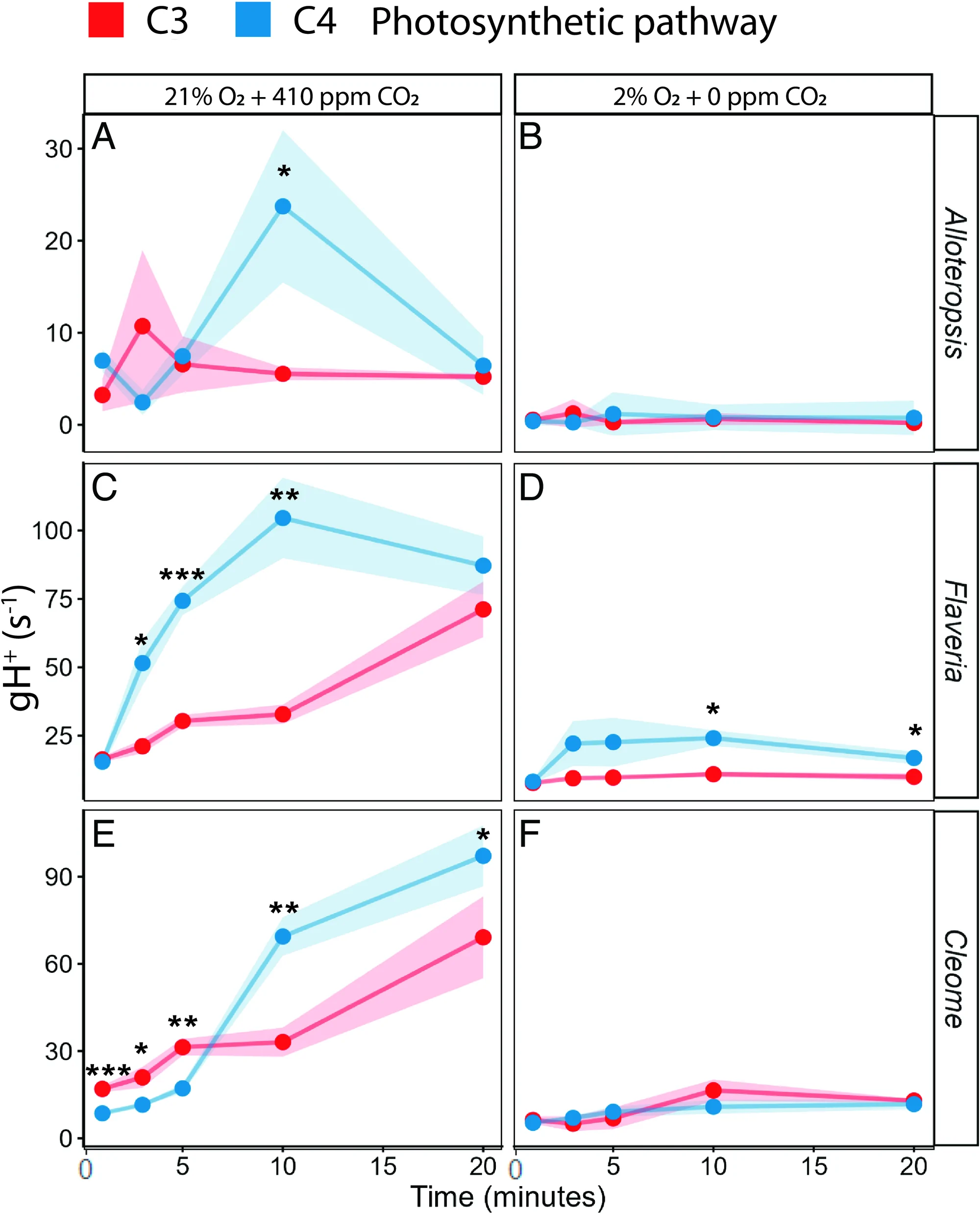
Proton conductivity of the thylakoid membrane (gH^+^) in C3 and C4 phylogenetically linked species of *Alloteropsis* (*A*, *B*), *Flaveria* (*C*, *D*) and *Cleome* (*E*, *F*). gH+ values were estimated from electrochromic shift measurements under ambient (*A*, *C*, *E*) and 2% O_2_ air and 0 ppm CO_2_ (*B*, *D*, *F*), during 20 min of illumination at 600 µmol m^−2^ s^−2^ PFD. Estimates of gH^+^ were obtained from Dark Induced Relaxation Kinetics as the inverse of the decay time constant (τ_ECS_) of a single exponential decay fitted to the first 300 ms of the dark interval. Asterisks indicate significant differences between species at a given timepoint found by one-way ANOVA (n = 5, *P ≤ 0.05, **P ≤ 0.01, ***P ≤ 0.001).

The higher rates of CEF to satisfy ATP:NADPH requirements (23) and larger electron sinks found in C4 photosynthesis (33) may contribute to the formation and collapse of ΔpH. To test whether fast NPQ relaxation in C4 species is linked to C4 metabolism, we repeated our NPQ measurements in 2% O_2_ + 0 ppm CO_2_ air (Fig. 4). The suppression of photosynthesis and photorespiration slowed down NPQ relaxation and removed differences between C3 and C4 *Alloteropsis* and *Cleome* pairs. Unlike the step-like decay observed in ambient air (Fig. 2*A*), NPQ in C4 *A. semialata MDG* and C4 *G. gynandra* followed the approximately exponential decay pattern of their C3 counterparts in 2% O_2_ + 0 ppm CO_2_ (Fig. 4*A*, values and statistics in *SI Appendix*, Table S1), and had similar relative contributions of the 0 to 2 min and 2 to 15 min components to total NPQ (Fig. 4*B* and *SI Appendix*, Table S2). In contrast, differences in NPQ kinetics between C3 and C4 species seemed enhanced in *Flaveria* in 2% O_2_ + 0 ppm CO_2_ air. Compared to C3 *F. cronquistii*, integrated NPQ in C4 *F. bidentis* was lower and the 0 to 2 min component still represented a significantly larger proportion of NPQ, while the 2 to 15 min component showed the opposite trend. Based on these results, we speculated that C4 *F. bidentis* may retain H^+^ efflux capacity in 2% O_2_ + 0 ppm CO_2_ air. To test this, we estimated gH^+^ from decay kinetics of electrochromic shift (ECS) signal measurements during brief dark intervals across 20 min of light to encompass the 0 to 2 and 2 to 15 min NPQ components (Fig. 5). Under ambient air, all C4 species tended to have higher gH^+^ than their C3 pairs (Fig. 5 *A, C,* and *E*). However, C4 *F. bidentis* retained H^+^ efflux capacity in 2% O_2_ + 0 ppm CO_2_ (Fig. 5*D*), albeit diminished compared to 21% O2 + 410 ppm CO_2_, whereas suppressing photosynthesis and photorespiration resulted in gH^+^ values approaching zero in all other species (Fig. 5 *B, D,* and *F*). These results show that fast NPQ relaxation in C4 species is strongly linked to proton efflux capacity and that an alternative electron sink in C4 *F. bidentis* likely sustains proton efflux and qE even when photorespiration and photosynthesis are suppressed.

### Evaluating CEF in C3 and C4 Species.

**Fig. 6.**
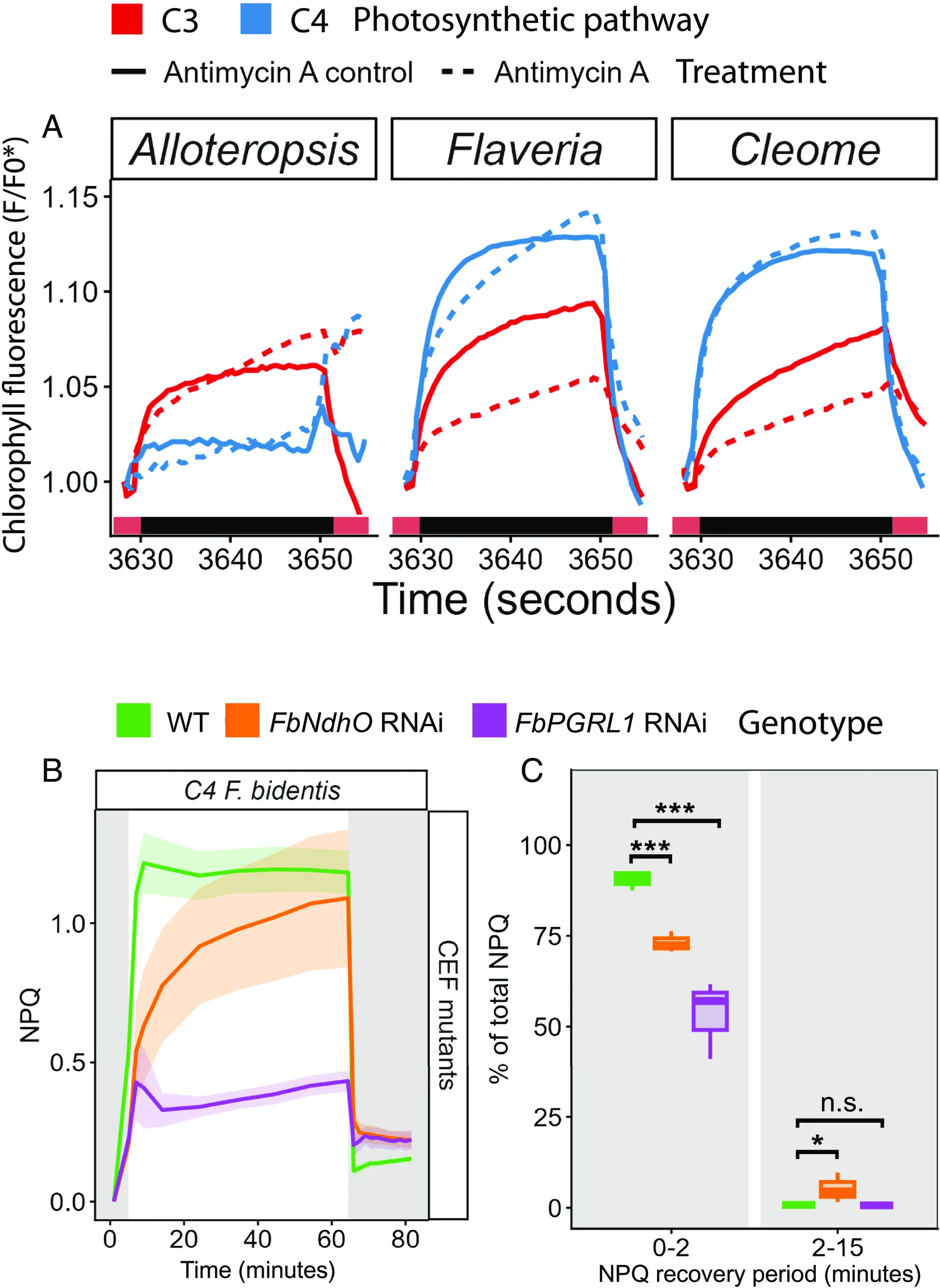
Analysis of NDH and PGR5/PGRL1-dependent CEF in C3 and C4 phylogenetically linked species. (*A*) Representative trace (n = 5) of PIFR in leaves infiltrated with a control buffer (solid lines) or with Antimycin A (dashed lines), an inhibitor of the CEF PGR5 pathway. (*B*) NPQ levels during 1 h of illumination at 600 µmol m^−2^ s^−2^ PFD followed by 25 min of darkness in C4 *F. bidentis* wild-type (WT) and *FbNdhO* and *FbPGRL1* RNAi knockdown mutants. Ribbons represent SE of the mean (n = 3). (*C*) NPQ composition based on time relaxation kinetics as a percentage of total NPQ. Asterisks indicate significant differences between each mutant and the WT found by one-way ANOVA (n = 3, *P ≤ 0.05, **P ≤ 0.01, ***P ≤ 0.001).

Chlorophyll fluorescence reflects the PSII-associated quinone redox state. In darkness, with no LEF-driven reduction, a postillumination chlorophyll fluorescence rise (PIFR) is attributed to residual CEF transferring electrons from stromal donors to the plastoquinone (PQ) pool that then equilibrates with PSII-associated quinones (55). We measured PIFR as a proxy for CEF after 1 h light treatment, periodically flashing far-red light to preferentially excite PSI and temporarily enhance PQ oxidation (full PIFR trace in *SI Appendix*, Fig. S4). After the far-red pulse, a larger PIFR response was observed in C3 *A. semialata KWT*, C4 *F. bidentis*, and C4 *G. gynandra* than in their phylogenetic pairs, indicating higher CEF activity in those species (Fig. 6*A*, solid lines). This is consistent with ATP:NADPH ratio requirements being higher in NADP-ME (C4 *F. bidentis*) and NAD-ME (C4 *G. gynandra*) subtypes than in mixed PEPCK pathways (C4 *A. semialata MDG*), which have lower ATP requirements (18). Leaves were also treated with PGR5/PGRL1 pathway inhibitor Antimycin A (dashed lines), which resulted in significantly lower PIFR in C3 *F. cronquistii* and C3 *T. hassleriana*, indicating a substantial contribution of PGR5/PGRL1 to PQ reduction. As with other infiltrations, various concentrations of Antimycin A were tested (*SI Appendix*, Fig. S3). The addition of Antimycin A also resulted in higher PIFR values in some cases—a secondary effect of Antimycin A that has been previously attributed to additional inhibition of electron transport downstream of PQ (31). Even with this effect, these results validate present understanding of C3 and C4 CEF pathways—C3 species primarily use the PGR5/PGRL1 route while C4 species use both the PGR5/PGRL1 and the NDH pathways (26, 30, 31). C3 *A. semialata KWT* appears to be an exception, but given that the C3 subspecies may have reversed from a C3 to C4 intermediate, CEF operation might differ from other C3 plants (56).

The role of CEF in C4 NPQ relaxation was further studied in C4 *F. bidentis FbPGRL1-RNAi* and *FbNdhO-RNAi* knockdown lines (31)—as previously observed, carbon assimilation was notably lower only in *FbNdhO-RNAi* (*SI Appendix*, Fig. S5), suggesting an impaired C4 cycle. NPQ in *FbNdhO-RNAi* decayed more slowly than the stepwise drop observed in WT and *FbPGRL1-RNAi* (Fig. 6*B*, values and statistics in *SI Appendix*, Table S5). While the 0 to 2 min component constituting most of WT NPQ was significantly reduced in both *FbPGRL1-RNAi* and *FbNdhO-RNAi*, the slower NPQ relaxation of *FbNdhO-RNAi* is evidenced by a more significant 2 to 15 min component than in WT. At the end of illumination, maximum NPQ was lower in *FbPGRL1-RNAi* than in WT and *FbNdhO-RNAi*, suggesting that differences in NPQ composition stem from impaired relaxation in *FbNdhO-RNAi*, but from overall NPQ suppression in *FbPGRL1-RNAi.*

## Discussion

### C4 Species Have Faster NPQ Relaxation.

This study sought to characterize differences in NPQ relaxation between C3 and C4 photosynthesis, by controlling for species variation at the genus level and comparing phylogenetically linked *Alloteropsis*, *Flaveria*, and *Cleome* C3 and C4 species. Despite considerable evolutionary distance between the three tested genera and mechanistic differences between the C4 pathways, all C4 species had significantly faster and overall greater NPQ relaxation than their C3 pairs (Fig. 2*A*). The meta-analysis of new and published C3 and C4 NPQ measurements in fourteen different species (Fig. 2*C* and *SI Appendix*, Table S3) confirms that faster NPQ is consistently found in C4 species, irrespective of growth conditions or light regimes. The rapid return of PSII to the unquenched state in C4 species would support higher photosynthetic quantum yields following decreases in irradiance and could be contributing to the more sustained rates of CO_2_ assimilation that have been observed in C4 vs. C3 species during light-shade transitions (36, 57). While increasing the rate of NPQ relaxation has been successful at improving photosynthetic efficiency in C3 species (9, 10), our results suggest that this approach may have less scope to stimulate carbon assimilation in C4 species given their intrinsically faster NPQ relaxation rate.

### qE Is a Greater Proportion of Total NPQ in C4 Species.

The comparatively faster NPQ relaxation found in C4 plants stemmed from differences in NPQ composition between C3 and C4 species. Separation of NPQ into components based on decay timescales revealed that C4 species had a significantly higher proportion of a component with a fast recovery period (0 to 2 min) compared to their C3 phylogenetic pairs, with C3 species exhibiting a comparatively greater proportion of NPQ relaxation within the 2 to 15 min timeframe (Fig. 2*B*). qE operates within the 0 to 2 min timescale, requires lumen acidification and is enhanced by zeaxanthin accumulation (2, 58). When infiltrated with nigericin, NPQ in C4 species was significantly more suppressed than in C3 species, whereas inhibiting xanthophyll cycle activity with DTT did not result in significant differences in the extent of NPQ reduction between C3 and C4 species (Fig. 3), consistent with faster NPQ identified in the C4 species being caused by an increased contribution of ∆pH-dependent qE. Activation of qE by lumen pH relies on the presence of photosystem II subunit S (PsbS), which initiates the quenched LHCII state via a conformational switch upon protonation of lumen-exposed protonatable residues (59, 60). Thus, one hypothesis could be that C4 species may show slight alterations in PsbS relative abundance, structure, or interactions with LHCs to explain the enhancement of qE. However, the faster NPQ kinetics observed in C4 species are not fully consistent with the NPQ phenotype of increased PsbS abundance. In PsbS-overexpressing lines of C3 species, qE enhancement concurs with significant increases in the amplitude of NPQ induction (61, 62), whereas the NPQ levels found in C4 species just before the light transition are not consistently higher than in their C3 pairs, with only C4 *F. bidentis* displaying an increased NPQ amplitude (Fig. 2*A* and *SI Appendix*, Fig. S1). In addition, overexpression of PsbS in the C4 species maize (50) resulted in more modest changes to the qE component than in C3 species (59, 61, 62) and crucially did not alter NPQ relaxation kinetics. Furthermore, if differences in PsbS accumulation underpinned the NPQ differences between C3 and C4 species, NPQ amplitude differences would be expected to show under 2% O_2_ + 0 ppm CO_2_ due to ∆pH buildup, yet no consistent increases in maximum NPQ were found in C4 species (*SI Appendix*, Fig. S4), with C4 *F. bidentis* even having lower NPQ induction than C3 *F. cronquistii*. Altogether, while we cannot fully rule out any impact of PsbS accumulation, faster NPQ relaxation in C4 species is more likely to be explained by altered regulation of lumen pH, specifically faster proton efflux, which in turn speeds up qE recovery.

### Faster Proton Efflux and NPQ Relaxation Are Linked to Altered Energetic Requirements of the C4 Pathway.

When photosynthesis and photorespiration were suppressed, NPQ relaxation in C4 *A. semialata MDG* and *G. gynandra* conformed to the slower exponential decay and composition of C3 *A. semialata KWT* and *T. hassleriana* (Fig. 4). In ambient air, C4 metabolism largely suppresses photorespiration so these results suggest that the fast NPQ relaxation observed in *Alloteropsis* and *Cleome* C4 species is specifically related to the ATP and NADPH demand by the C4 photosynthetic pathway. qE is regulated by lumen pH, which is in turn dependent on membrane proton conductivity, gH^+^ (32). The higher gH^+^ found in all three C4 species in ambient conditions (Fig. 5 *A, C,* and *E*) provides a mechanistic explanation underpinning accelerated relaxation of pH-dependent NPQ, primarily qE. Different C4 pathways have distinct ATP:NADPH requirements: In NADP-ME/PEPCK subtypes (C4 *A. semialata MDG*), the energetic balance is comparable to C3 species, whereas NADP-ME (*F. bidentis*) and NAD-ME (*G. gynandra*) pathways have elevated ATP:NADPH demands. The increased ATP demand is localized in BS cells in NADP-ME subtypes but in M cells in NAD-ME subtypes (16–18). M cells are positioned in the outer layer of Kranz anatomy and therefore represent most of the chlorophyll fluorescence signal used to determine NPQ. However, in all three C4 species, the large metabolic pools of C4 cycle intermediates required to sustain diffusion gradients between M and BS cells may underpin the observed increases in gH^+^ relative to C3 species. The high gH^+^ indicates upregulated proton efflux, reflecting activity of ATP synthase (32, 63) as well as a contribution of thylakoid antiporters like KEA3 (64). Whereas both mechanisms could be upregulated in C4 photosynthesis, the former seems consistent with the increased ATP demand of the C4 pathway. Uniquely, in C4 *F. bidentis* NPQ relaxation was not slowed by the suppression of photosynthesis and photorespiration (Fig. 4), and gH^+^ remained higher than in the other C4 species (Fig. 5*D*), indicating the presence of an alternative electron sink. The identity of this electron sink remains unclear, but it seems plausible that it relates to specific attributes of the canonical NADP-ME C4 pathway, such as the significantly larger malate pool sizes that could go toward ATP-consuming metabolic reactions (65).

### Contribution of CEF Pathways to Faster NPQ in C4 Species.

In C3 species, CEF via the PGR5/PGRL1 pathway is essential for photoprotection by contributing to ∆pH formation and qE (24, 25). Relative to the ancestral C3 pathway, PGR5/PGRL1 abundance increases in C4 species in both M and BS cells (17, 66), while NDH differentially accumulates per ATP requirements: in M cells for NAD-ME pathways and in BS for NADP-ME (16, 18). Our results point to both NDH and PGR5/PGRL1 pathways contributing to fast relaxation in C4 NPQ. Both C4 *FbNdhO-RNAi* and *FbPGRL1-RNAi* lines showed altered NPQ relaxation kinetics relative to WT (Fig. 6*B*). Consistent with our findings, previous work found the PGR5/PGRL1 pathway to significantly contribute to NPQ induction in C4 *F. bidentis* (31). Here, we also show that knocking down PGRL1 results in a specific decrease in qE activation (Fig. 6*C*), probably due to deficient *pmf* formation. In contrast, reduction of NDH expression primarily slowed down NPQ responses. C4 *F. bidentis* NDH is primarily found in the BS, accounting for most of BS CEF (30). Although the chlorophyll fluorescence signal primarily comes from M chloroplasts, an impaired 0 to 2 min NPQ component was still observed in *FbNdhO-RNAi* mutants (Fig. 6*C*). The tissue-specific expression of NDH in C4 species and the deleterious effect of its suppression on growth and carbon assimilation suggests NDH is the major route for ATP provision to C4 metabolism (30, 31). Accordingly, the rate of carbon assimilation in *FbNdhO-RNAi* was greatly reduced relative to wild type (*SI Appendix*, Fig. S4). An impaired C4 cycle in *FbNdhO-RNAi* would alter inorganic phosphate availability and LEF:CEF energy balance, potentially diminishing CEF-related qE and leading to parallel decreases in gH^+^ and ∆pH in M cells (32, 33). Since the role of NDH in C4 photosynthesis has thus far only been studied in NADP-ME plants with NDH-enriched BS, it remains unclear if NDH-mediated CEF generally also plays a photoprotective role similar to PGR5/PGRL1. Future research could test this in NAD-ME species with NDH-enriched M (22), such as C4 *G. gynandra*.

The complexity of NPQ molecular mechanisms, evolution of CEF pathways, and biochemical diversity within C4 species leave many outstanding questions with relation to C4 NPQ. Nevertheless, the results presented here demonstrate that the higher rates of CEF and larger electron sinks of C4 metabolism result in enhancement of the qE component, leading to faster relaxation kinetics. The increased contribution of qE to total NPQ in C4 species mimics the engineering attempts to accelerate NPQ responses in C3 crops via single overexpression of PsbS (61, 67) or in combination with VDE and zeaxanthin epoxidase (9, 10), which may therefore have less scope to further accelerate NPQ responses and photosynthetic efficiency in C4 species.

## Materials and Methods

### Plant Material and Growth Conditions.

C4 *F. bidentis*, C3 *F. cronquistii*, C4 *G. gynandra,* and C3 *T. hassleriana* were grown in controlled environment conditions at 20 °C and 150 µmol m^−2^ s^−1^ PFD over a 16-h photoperiod. *A. semialata MDG* and *A. semialata KWT* were grown in a 4:1 mix of soil and vermiculite under semicontrolled glasshouse conditions at 18 to 25 °C with supplemental lighting provided to a minimum of 140 to 160 µmol m^−2^ s^−1^ PFD over a 16-h photoperiod (further detail in ref. 35). Measurements were conducted on fully expanded leaves during vegetative stage: at 8 to 10 wk for both *Flaveria* species and *G. gynandra*, 4 to 6 wk for *T. hassleriana*, and 2 wk after vegetative propagation for both *Alloteropsis* species. C4 *F. bidentis* WT, *FbPGRL1-RNAi,* and *FbNdhO-RNAi* RNAi plants with expression ~10% of WT (31), were grown in a 3:2 mix of soil and vermiculate, in a growth chamber at 24 °C and 250 µmol m^−2^ s^−1^ PFD over a 12-h photoperiod. Young, fully expanded *FbNdhO-RNAi* leaves were measured after 12 to 16 wk and of all other plants after 8 to 10 wk.

For additional chlorophyll fluorescence measurements of C3 and C4 species, *Setaria viridis* and Kitaake rice (*O. sativa* ssp. *japonica*) were grown in a 1:1 mix of topsoil and sand in a growth cabinet at 28 °C, 65% relative humidity, and 400 µmol m^−2^ s^−1^ PFD over a 12-h photoperiod. The youngest fully expanded leaves of 20-d-old plants were measured. *Arabidopsis thaliana* seeds were planted in a 4:1 mix of soil and sand, stratified at 4 °C for 3 d, then grown in a chamber at 20 °C, 60% relative humidity, and 200 µmol m^−2^ s^−1^ PFD over an 8-h photoperiod, with measurements conducted on 7 to 9-wk-old plants. Sugarcane (*Saccharum officinaum*, CPCL02-0926) and American and African soybean (*Glycine max*, LD11 and TGx2014-16FM) were measured 27 d after being sown in soil under controlled conditions on a 14-h light (26 °C)/10-h dark (23 °C) cycle, with relative humidity maintained between 55% and 60%. During the photoperiod, light alternated every 20 min between 1,800 µmol m^–2^ s^–1^ and 200 µmol m^–2^ s^–1^. Maize (*Zea maize*, HC69) was grown in soil in a greenhouse in Illinois at 22 to 28 °C and the fifth leaf was measured 5 wk after planting. Finally, *Marchantia polymorpha* was grown on 0.5 Gamborg’s B5 medium with vitamins and 1.2% agar, at 21 °C under 150 µmol m^–2^ s^–1^ continuous light and measured 1 wk after germination.

### Chlorophyll Fluorescence Measurements.

For measurements conducted on phylogenetically linked species, chlorophyll fluorescence was measured with a gas exchange system (LI-6400XT, LI-COR, Lincoln, NE) equipped with a leaf chamber fluorometer (6400-40 LCF, LI-COR). Chamber conditions were controlled at 410 or 0 ppm CO_2_ concentration (the latter with 2% O_2_ air), 40 to 60% relative humidity, 25 °C block temperature, 300 µmol s^−1^ flow rate, and 10% blue (470 nm) and 90% red actinic light (630 nm). The LCF used a 0.25 kHz modulated measuring light and a multiphase flash (68) to measure chlorophyll fluorescence parameters.

Leaves were dark-adapted until stomatal conductance and net CO_2_ exchange rate stabilized (30 to 60 min), illuminated with 600 or 1,500 µmol m^−2^ s^−1^ PFD for 1 h, and then returned to darkness for 25 min. Light intensities were selected as saturating or excess light based on species-specific AQ curves (35); subsequent experiments focused on the saturating treatment to avoid photodamage as a confounding factor. Multiphase saturating flashes with phase 1 and 3 intensity at 4,000 µmol m^−2^ s^−1^ PFD were used to measure steady and maximal fluorescence after dark-adaption (*F* and *F_m_*), and during photoperiod and dark recovery (*F′* and *F_m_′*), occurring 5 min before illumination (for *F_v_/F_m_*), 3, 5, 10, 15, 25, 35, 45, and 60 min into light exposure, 30 s after returning to dark, and every 90 s during subsequent dark recovery. NPQ was derived from *F_m_* and *F_m_*′ measurements (69). NPQ relaxation was compared by calculating integrated NPQ from area under the curve (AUC); and 0 to 2 min and 2 to 15 min component contributions were expressed as the integrated NPQ within each phase relative to total postillumination NPQ. To suppress photosynthesis and photorespiration, a premixed 2% O_2_ and 98% N_2_ gas mixture (BOC Ltd., Woking, UK) was supplied to the gas exchange system air inlet using a mass flow controller (EL-FLOW, Bronkhorst Hight-tech BV, Ruurlo, NL). Chlorophyll fluorescence rise during dark recovery [following (55)] was monitored for 2.5 min in darkness after illumination, interspersed by 5 s of far-red (FR) light to oxidize the PQ pool (740 nm, ~50 μmol of photons m^−2^ s^−1^) every 20 to 25 s. We compared the PIFR of the dark period after FR, normalized to the final fluorescence value postoxidation (F0*, see representative full traces in *SI Appendix*, Fig. S4).

Additional measurements on other C3 and C4 species were conducted with the above system (rice, maize, *Setaria*); or with a chlorophyll fluorescence imager on leaf disks (CFImager, Technologica Ltd., UK, for soybean and sugarcane), or whole leaves (FluorCam FC 800-C, PSI, Czech Republic, for *Arabidopsis* and *Marchantia*). After dark adaption, NPQ measurements were taken every minute with two protocols that complemented existing studies: 50 µmol m^−2^ s^−1^ PFD for 5 min, 1,500 µmol m^−2^ s^−1^ PFD for 15 min and 50 µmol m^−2^ s^−1^ PFD for 45 min; and 10 min each of µmol m^−2^ s^−1^ PFD and darkness (see *SI Appendix*, Table S3 for all experimental conditions for each species).

### Nigericin, DTT, and Antimycin A Leaf Infiltration.

Dark-adapted leaves were vacuum infiltrated with NPQ inhibitors using a syringe with buffer (20 mM HEPES/KOH pH 7.0) containing 100 µM nigericin, 5 mM DTT, or 250 µM Antimycin A. Controls were infiltrated with buffer and equivalent volume of solvent. While these inhibitors are commonly used to manipulate pH gradients, VDE activity, and PGR5/PGRL1-dependent cyclic electron flow, respectively, they all have potential side-effects. To minimize these, various concentrations were tested in a shortened assay to find a concentration with limited off-target effects (*SI Appendix*, Fig. S3). The concentrations used for analysis were determined from a dose–response experiment wherein leaves were infiltrated with increasing concentrations of each chemical and measured under a 20-min photoperiod (*SI Appendix*, Fig. S3). Since antimycin A also inhibits respiratory cytochrome C reductase, dark respiration of infiltrated leaves relative to controls were also checked prior to measuring. Following infiltration, leaves were kept in darkness for a further 20 min before measuring. The effect of NPQ inhibitors was estimated by calculating NPQ AUC during the light period and expressing as a proportion of control NPQ AUC (*SI Appendix*, Fig. S2 for full NPQ traces).

### Electrochromic Shift Measurements.

The ECS signal was measured as absorptance changes at 515 nm using 535 nm as an isosbestic waveband (Dual-KLAS fitted with a P515/535 module, Heinz Walz GmbH, Effeltrich, DE) (70). The fore-optics were integrated in a custom measuring chamber (GFS-3000 measuring chamber for DUAL-KLAS, Walz) with temperature controlled at 25 °C. Chamber conditions were controlled via the console of a LI-6800 gas exchange system (LI-COR, United States) at 410 or 0 ppm sample CO_2_ concentration (the latter with 2% O_2_ air), 60% relative humidity, and 200 µmol s^−1^ flow rate. Leaves were dark-adapted and illuminated with 600 µmol m^−2^ s^−1^ PFD for 20 min. Dark Interval Relaxation Kinetics (DIRK) measurements of the ECS signal (71) were taken at 1, 3, 5, 10, and 20 min of illumination. Estimates of thylakoid membrane proton conductivity (gH^+^) were obtained from the inverse of the decay time constant (τ_ECS_) of a single exponential decay fitted to the first 300 ms of the dark interval (72).

### Phylogenetic Tree Visualization.

Phylogenetic relationships among angiosperm families (73), and between species within the same families included in this study, were visualized using a synthetic tree derived from the Open Tree of Life (74) and edited with iTOL (75) to show C4 origins and species distribution (45).

### Meta-Analysis of NPQ Relaxation across C3 and C4 Species.

NPQ induction and relaxation measurements of WT C3 and C4 species were obtained from *SI Appendix* of published studies (9, 48–54). To supplement these data, additional measurements were taken on rice, soybean, maize, sugarcane, *M. polymorpha, A. thaliana,* and *S. viridis.* The final species list thus included C3 tomato, rice, tobacco, *Marchantia*, barley, soybean, and *Arabidopsis*; and C4 maize, sorghum, *Setaria*, sugarcane, *Miscanthus sacchariflorus, M. giganteus,* and *M. sinensis*. Given differences in light periods and relaxation timelines, only the first 15 min of NPQ relaxation were fitted with a single exponential model, from which relaxation constant *k* was obtained. Each individual datapoint was fitted separately and then averaged, and only those averages were then plotted and analyzed to prevent experiments with large datasets (48, 49, 51, 53) from biasing the data.

### Statistical Analysis.

One-way ANOVA was conducted on independent experiments comparing each C3 and C4 phylogenetic pair, relaxation constants of C3 and C4 species, and comparing *FbPGRL1-RNAi* and *FbNdhO-RNAi* mutants with WT. Assumptions of normality and homogeneity of variance were tested for, respectively, with Shapiro–Wilk and Bartlett’s tests.

## Supplementary Material

Appendix 01 (PDF)

## Data Availability

All shared data are included in the article and/or *SI Appendix*.
