# Supplementary material for: Faster relaxation of nonphotochemical quenching in C4 than in C3 species
Source: Proc Natl Acad Sci U S A. 2026 Sep 18;123(38):e2621033123. doi: 10.1073/pnas.2621033123 (PMC13598202; doi:10.1073/pnas.2621033123)
Supplement: Supplementary file 1 [file pnas.2621033123.sapp.pdf]

Table S1: Values and statistics of integrated NPQ across dark recovery in phylogenetic pairs. Integrated NPQ and standard error of the mean across dark recovery period in C3 and C4 species during the 25 min darkness following 1h of illumination at 600 µmol m^{-2} s^{-2} PFD, in ambient and 2% O_2 and 0 ppm CO_2 air (n=5). One-way ANOVA was conducted between phylogenetically linked species (degrees of freedom; *F*-value; *p*-value), significant *p*-values are shown in bold.

Experiment	Species	Integrated NPQ	ANOVA
Ambient conditions	C4 *A. semialata MDG*	11.59±1.50	1,8; 5.85; **0.049**
	C3 *A. semialata KWT*	19.21±2.54	
	C4 *F. bidentis*	8.35±0.68	1,8; 17.72; **0.003**
	C3 *F. cronquistii*	12.45±0.73	
	C4 *G. gynandra*	11.63±.96	1,8; 6.14; **0.042**
	C3 *T. hassleriana*	14.86±0.74	
2% O_2 + 0 ppm CO_2	C4 *A. semialata MDG*	22.29±3.06	1,8; 0.225; 0.65
	C3 *A. semialata KWT*	23.81±0.99	
	C4 *F. bidentis*	10.93±1.90	1,8; 87.09; **≤0.001**
	C3 *F. cronquistii*	32.81±1.38	
	C4 *G. gynandra*	14.39±1.01	1,8; 1.26; 0.28
	C3 *T. hassleriana*	16.31±1.39	

Table S2: Values and statistics of NPQ composition in phylogenetic pairs. NPQ composition based on time relaxation kinetics as a percentage of total NPQ with standard error of the mean in C3 and C4 species, in ambient and 2% O_2 and 0 ppm CO_2 air (n=5). One-way ANOVA was conducted between phylogenetically linked species for each component (degrees of freedom; *F*-value; *p*-value), significant *p*-values are shown in bold.

Experiment	Component	Species	Component of total NPQ (%)	ANOVA
Ambient conditions	0-2 minutes	C4 *A. semialata MDG*	77±3	1,8; 5.77; **0.047**
		C3 *A. semialata KWT*	64±3	
		C4 *F. bidentis*	87±3	1,8; 31.58; **≤ 0.001**
		C3 *F. cronquistii*	47±6	
		C4 *G. gynandra*	78±1	1,8; 7.56; **0.028**
		C3 *T. hassleriana*	68±3	
	2-15 minutes	C4 *A. semialata MDG*	10±2	1,8; 5.80; **0.049**
		C3 *A. semialata KWT*	19±2	
		C4 *F. bidentis*	0±0	1,8; 98.65; **≤0.001**
		C3 *F. cronquistii*	28±3	
		C4 *G. gynandra*	3±1	1;8, 11.31; **0.012**
		C3 *T. hassleriana*	13±3	
2% O_2 + 0 ppm CO_2	0-2 minutes	C4 *A. semialata MDG*	66±4	1,8; 4.01; 0.061
		C3 *A. semialata KWT*	60±1	
		C4 *F. bidentis*	86±2	1,8; 4.72; **≤0.001**
		C3 *F. cronquistii*	31±1	
		C4 *G. gynandra*	60±1	1,8; 2.39; 0.14
		C3 *T. hassleriana*	59±1	
	2-15 minutes	C4 *A. semialata MDG*	20±2	1,8; 1.92; 0.18
		C3 *A. semialata KWT*	22±2	
		C4 *F. bidentis*	2±1	1,8; 357.03; **≤0.001**
		C3 *F. cronquistii*	27±1	
		C4 *G. gynandra*	13±1	1,8; 2.51; 0.13
		C3 *T. hassleriana*	15±1	

Table S3: NPQ relaxation constant of diverse C3 and C4 species. Values, statistics, and experimental descriptions of NPQ data from new and published measurements of C3 and C4 species covering a breadth of growth and experimental conditions. Relaxation constant *k* was obtained by fitting a single exponential model to NPQ relaxation in darkness or low light. Table shows the average relaxation constant for each study with standard error of the mean, plus the number of observations measured. All data utilised for the meta-analysis came from intact leaves and wild-type plants, and growth conditions in growth chambers or fields varied across studies, as did stage of measurement. A one-way ANOVA was conducted between C3 and C4 species averages (degrees of freedom; *F*-value; *p*-value), the significant *p*-value is shown in bold.

Photosynthetic pathway	Species	Experimental conditions	Relaxation constant *k* (min^{-1})	n	ANOVA
C3	Tomato (*Solanum lycopersicum*)	10 minutes 1500 μmol m^{-2} s^{-2} PFD and 12 minutes of darkness (53).	0.51±0.04	9	1,20; 6.063; **0.023**
	Rice (*Oryza sativa*)	8 minutes 1800 μmol m^{-2} s^{-2} PFD and 3 minutes of darkness (49).	1.45±0.57	3	
		5 minutes 50 μmol m^{-2} s^{-2} PFD, 15 minutes 1600 μmol m^{-2} s^{-2} PFD and 45 minutes 50 μmol m^{-2} s^{-2} PFD (new data).	0.42±0.04	5	
		10 mins 1500 μmol m^{-2} s^{-2} PFD and 10 mins of darkness (new data).	0.57±0.03	5	
	Tobacco (*Nicotiana tabacum)*	10 minutes 1000 μmol m^{-2} s^{-2} PFD and 10 minutes of darkness (9).	1.90±0.04	18	
	Marchantia polymorpha	10 minutes 1500 μmol m^{-2} s^{-2} PFD and 10 minutes of darkness (new data).	1.49±0.13	3	
	Barley (*Hordeum vulgare*)	10 minutes 1500 μmol m^{-2} s^{-2} PFD and 12 minutes of darkness (53).	1.00±0.11	6	
	Soybean (*Glycine max)*	5 minutes 50 μmol m^{-2} s^{-2} PFD, 15 minutes 1600 μmol m^{-2} s^{-2} PFD and 45 minutes 50 μmol m^{-2} s^{-2} PFD (new data).	0.45±0.01	5	
		Diversity panel, 20 minutes 50 μmol m^{-2} s^{-2} PFD, 15 minutes 2000 μmol m^{-2} s^{-2} PFD, 50 minutes 50 μmol m^{-2} s^{-2} PFD (48).	0.49±0.01	412	
	Arabidopsis thaliana	10 minutes 1500 μmol m^{-2} s^{-2} PFD and 10 minutes of darkness (new data).	1.42±0.13	15	
C4	Maize (*Zea mays*)	Diversity panel, 10 minutes 2000 μmol m^{-2} s^{-2} PFD and 20 minutes of darkness (52).	2.29±0.03	3057	
		10 minutes 1500 μmol m^{-2} s^{-2} PFD and 12 minutes of darkness (53).	2.06±0.13	4	
		8 minutes 1800 μmol m^{-2} s^{-2} PFD and 3 minutes of darkness (49).	2.24±0.05	3	
		5 minutes 50 μmol m^{-2} s^{-2} PFD, 15 minutes 1600 μmol m^{-2} s^{-2} PFD and 45 minutes 50 μmol m^{-2} s^{-2} PFD (new data).	0.93±0.08	5	
	Sorghum (*Sorghum bicolor)*	Diversity panel, 10 minutes 2000 μmol m^{-2} s^{-2} PFD and 10 minutes of darkness (50).	3.06±0.03	1932	
		Diversity panel, 10 minutes 2000 μmol m^{-2} s^{-2} PFD and 10 minutes of darkness (47).	4.89±0.08	1550	
	Setaria viridis	5 minutes 50 μmol m^{-2} s^{-2} PFD, 15 minutes 1600 μmol m^{-2} s^{-2} PFD and 45 minutes 50 μmol m^{-2} s^{-2} PFD (new data).	0.53±0.02	5	
		10 minutes 1500 μmol m^{-2} s^{-2} PFD and 10 minutes of darkness (new data).	1.61±0.04	5	
	Sugarcane (*Saccharum officinaum)*	5 minutes 50 μmol m^{-2} s^{-2} PFD, 15 minutes 1600 μmol m^{-2} s^{-2} PFD and 45 minutes 50 μmol m^{-2} s^{-2} PFD (new data).	0.71±0.14	5	
	Miscanthus (*Miscanthus sacchariflorus, Miscanthus giganteus, Miscanthus sinensis*)	10 minutes 2000 μmol m^{-2} s^{-2} PFD and 10 minutes of darkness (51).	1.71±0.06, 1.64±0.10, 1.87±0.16	11, 10, 8	

Table S4: Values and statistics of NPQ with chemical inhibitors in phylogenetic pairs. Integrated NPQ and standard error of the mean in C3 and C4 species infiltrated with 100 μM nigericin to collapse the proton gradient or 5 mM dithiothreitol to inhibit the xanthophyll cycle, as a percentage of a control across 1h of illumination at 600 μmol m^{-2} s^{-2} PFD (n=5). One-way ANOVA was conducted between phylogenetically linked species (degrees of freedom; *F*-value; *p*-value), significant *p*-values are shown in bold.

Inhibitor	Species	Integrated NPQ relative to control (%)	ANOVA
100 μmol Nigericin	C4 *A. semialata MDG*	25±3	1,8; 5.52; **0.05**
	C3 *A. semialata KWT*	42±9	
	C4 *F. bidentis*	12±2	1,8; 26.31; **≤0.001**
	C3 *F. cronquistii*	38±4	
	C4 *G. gynandra*	6±3	1,8; 6.80; **0.03**
	C3 *T. hassleriana*	22±6	
5 mM DTT	C4 *A. semialata MDG*	42±5	1,8; 1.90; 0.22
	C3 *A. semialata KWT*	34±3	
	C4 *F. bidentis*	59±6	1,8; 0.39; 0.55
	C3 *F. cronquistii*	51±10	
	C4 *G. gynandra*	59±5	1,8; 0.56; 0.47
	C3 *T. hassleriana*	53±5	

Table S5: Values and statistics of NPQ composition in CEF mutants. NPQ composition based on time relaxation kinetics as a percentage of total NPQ with standard error of the mean in C4 *F. bidentis* CEF mutants (n=3). One-way ANOVA was conducted between each mutant and the wild type for each component (degrees of freedom; *F*-value; *p*-value), significant *p*-values are shown in bold.

Component	Genotype	Component of total NPQ (%)	ANOVA with WT
0-2 minutes	WT	90±1	//
	FbNndhO RNAi	73±2	1,6; 94.26; **≤0.001**
	FbPGRL1 RNAi	53±6	1,6; 61.46; **≤0.001**
2-15 minutes	WT	-3±1.2	//
	FbNndhO RNAi	5±2	1,6; 13.85; **0.01**
	FbPGRL1 RNAi	-3±2	1,6; 0.09; 0.78

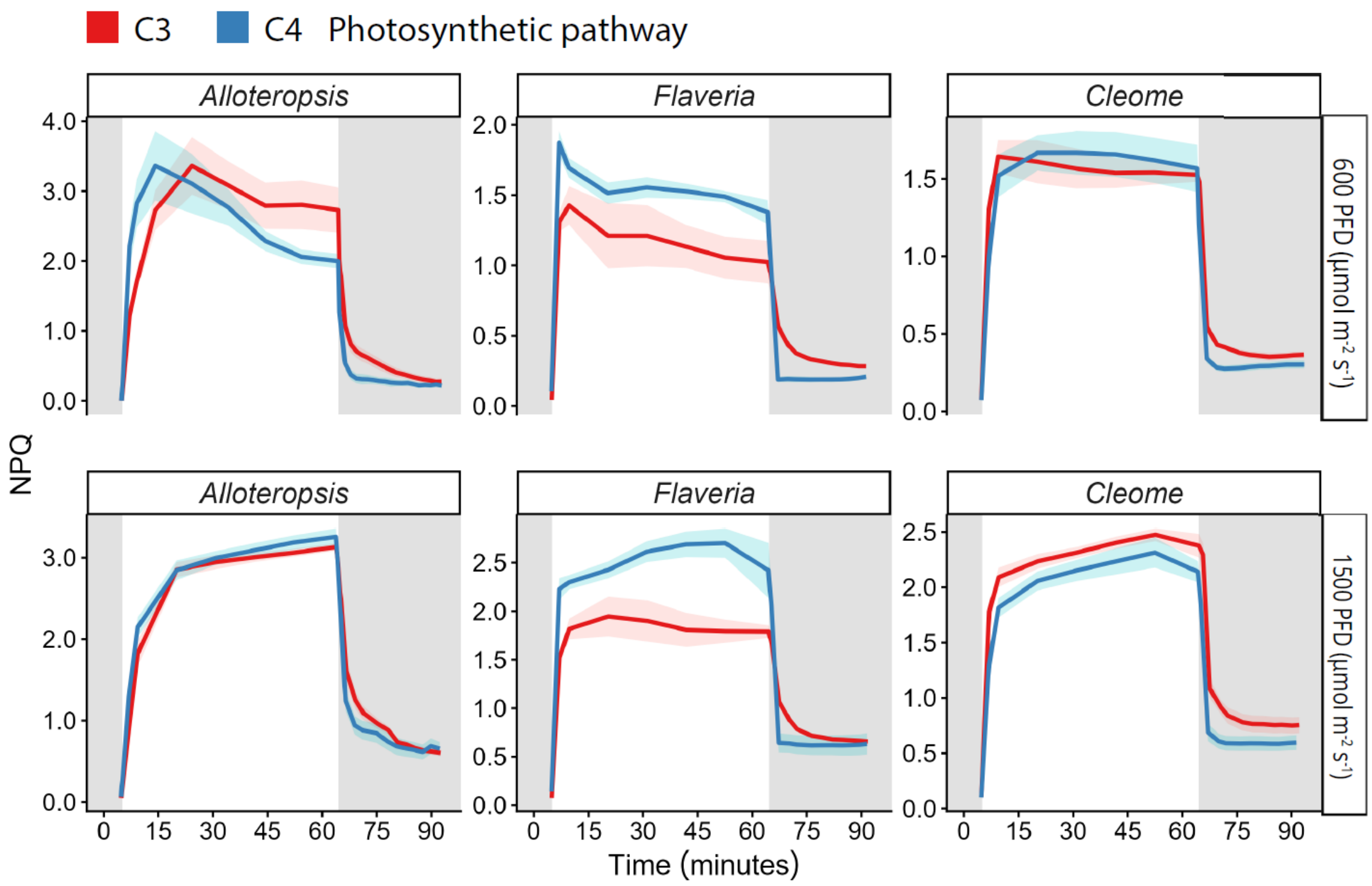

Fig. S1: NPQ induction and relaxation in phylogenetic pairs. NPQ induction and relaxation in phylogenetically linked C3 and C4 species during 1h of illumination at 600 µmol m^{-2} s^{-2} PFD or 1500 µmol m^{-2} s^{-2} PFD followed by 25 min of darkness. Ribbons represent standard error of the mean (n=5).

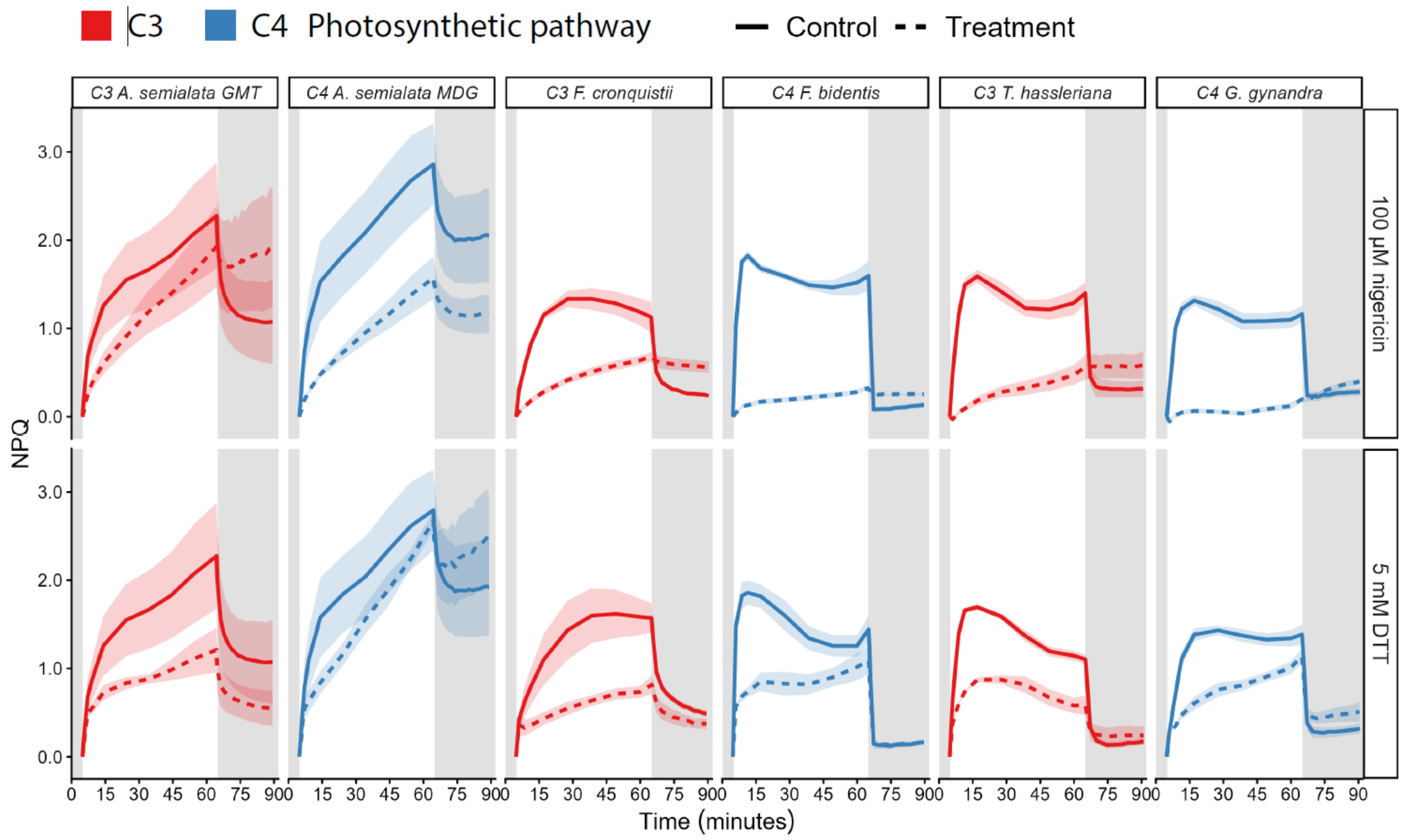

Fig. S2: NPQ induction and relaxation with chemical inhibitors. NPQ induction and relaxation during 1h of illumination at 600 µmol m^{-2} s^{-2} PFD followed by 25 min of darkness in C3 and C4 species infiltrated with a control buffer (solid lines); 100 µM nigericin to collapse the proton gradient, or 5 mM dithiothreitol to inhibit the xanthophyll cycle (dashed lines). Ribbons represent standard error of the mean (n=5).

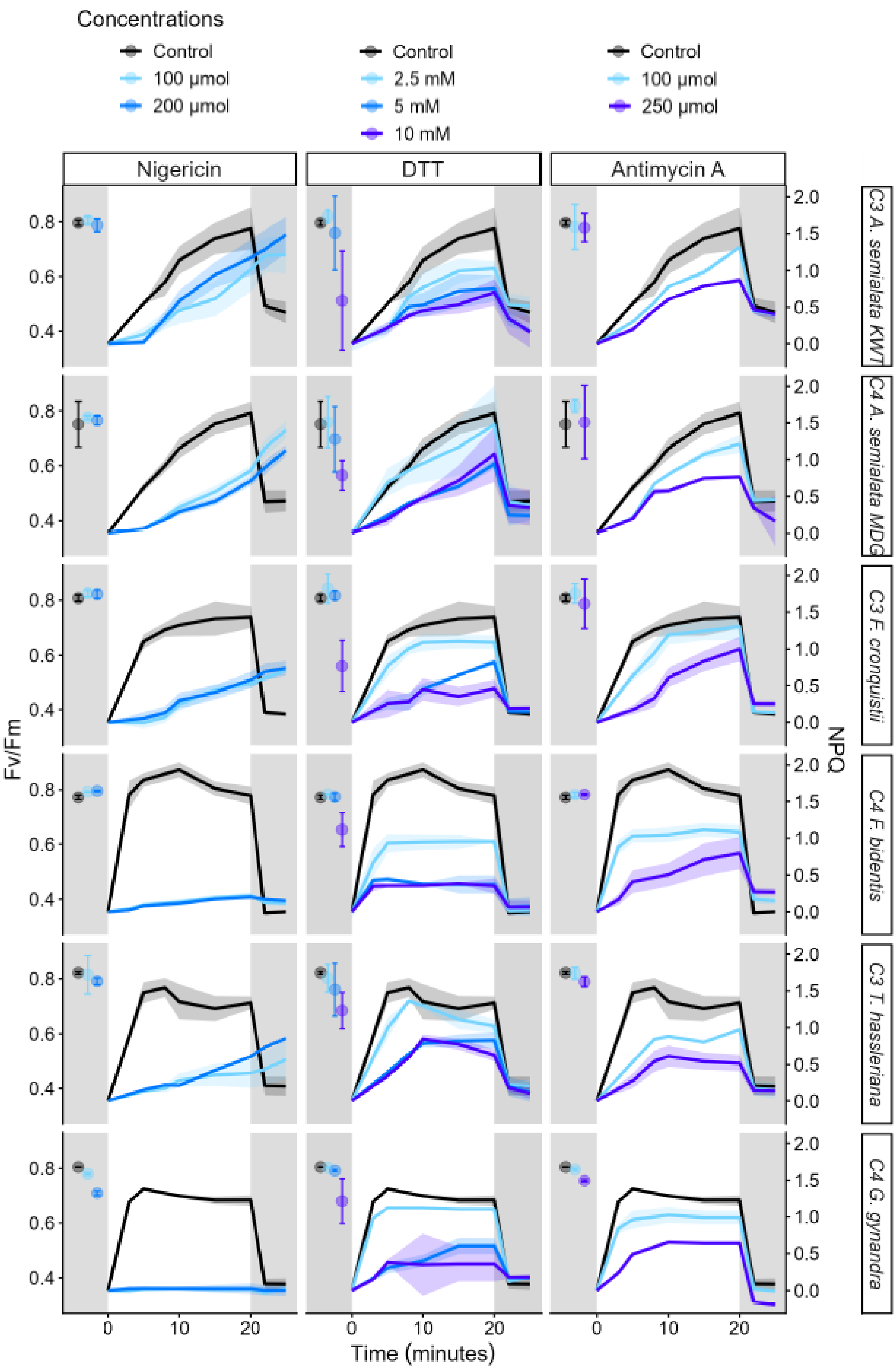

Fig. S3: Chemical infiltrations at diverse concentrations. Effects of infiltrating leaves with diverse concentrations of nigericin, DTT, and Antimycin A on F_v/F_m (circles) and NPQ across 20 min of illumination at 600 µmol m^{-2} s^{-2} PFD followed by 10 min of dark relaxation. Error bars and ribbons represent standard error of the mean (n=3).

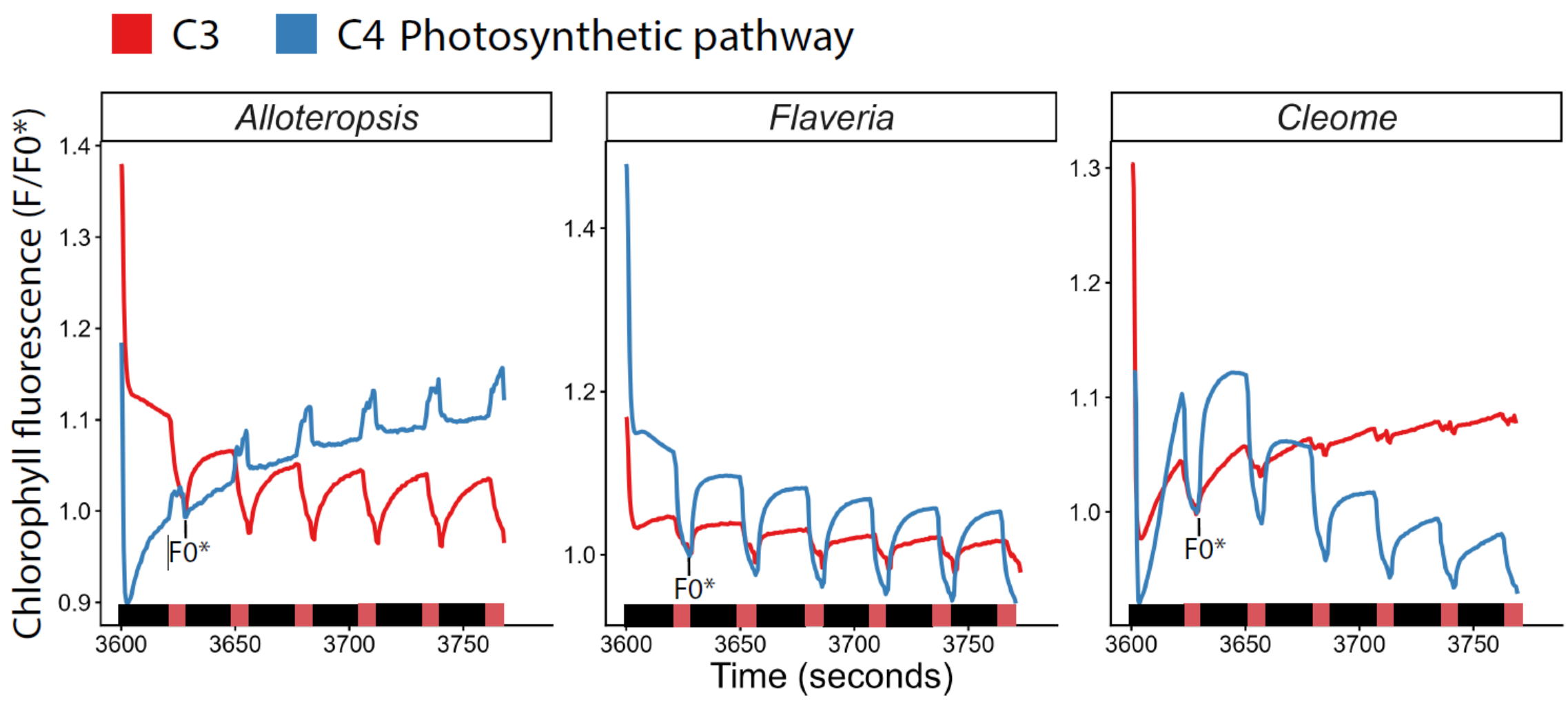

Fig. S4: Sample full trace of Post-Illumination Fluorescence Rise. Representative full traces of Post Illumination Fluorescence Rise measurements in C3 and C4 phylogenetically paired species after 1h of illumination at 600 µmol m^{-2} s^{-2} PFD. F' was measured during 20-25s dark intervals from instrument variation interspersed by 5s of far-red light. The fluorescence trace was normalised to *F0**, the final fluorescence value after the first pulse of far-red light (indicated in figure).

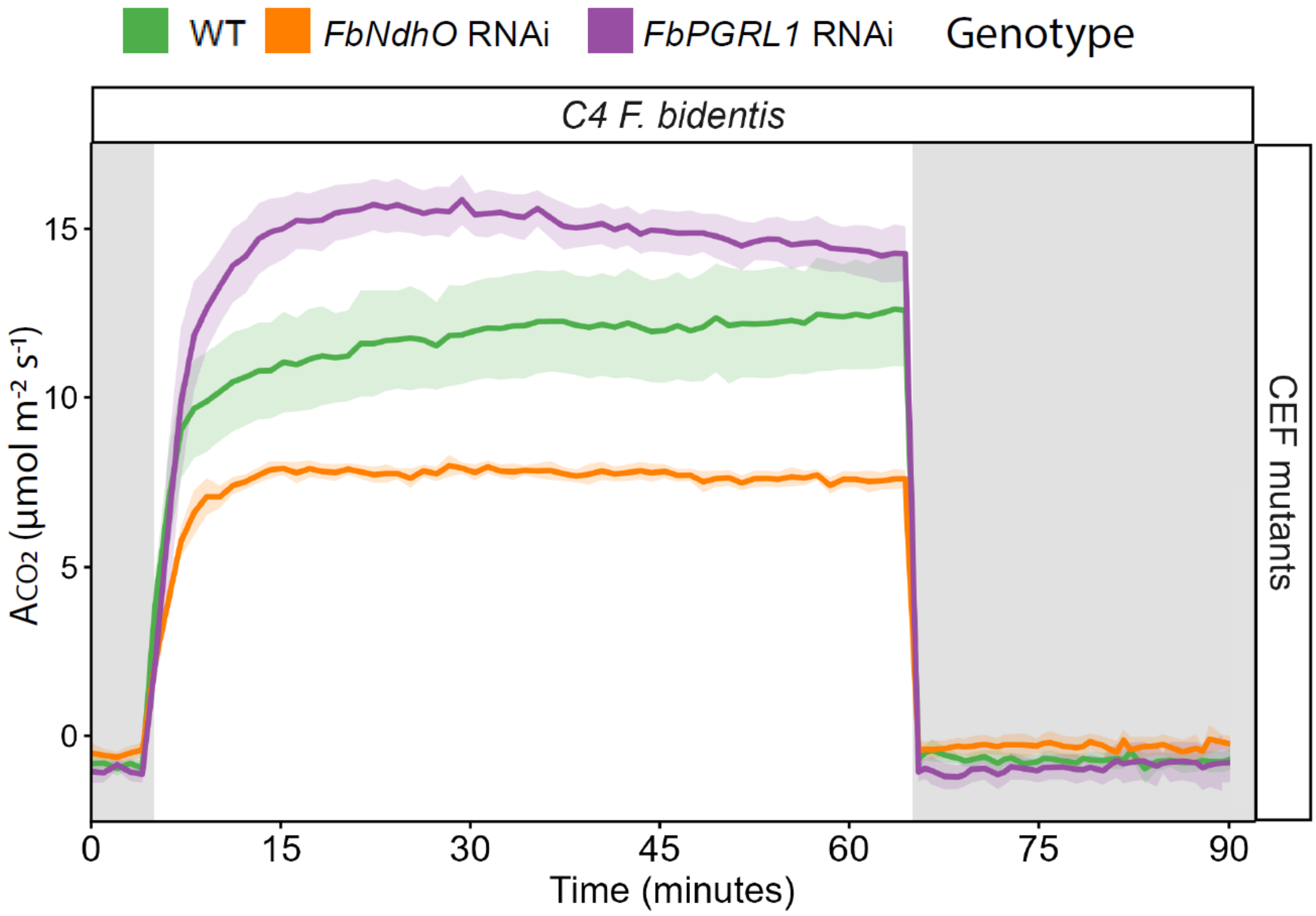

Fig. S5: Carbon assimilation of CEF mutants. Carbon assimilation in WT and CEF mutants of C4 *F. bidentis* during 1h of illumination at 600 µmol m^{-2} s^{-2} PFD preceded by 5 min and followed by 25 min of darkness. Ribbons represent standard error of the mean (n=3).
